# Prevalence of Depression in Patients with Juvenile Idiopathic Arthritis Presenting at a Tertiary Care Hospital

**DOI:** 10.7759/cureus.6807

**Published:** 2020-01-28

**Authors:** Saira Bano, Khalid Bosan, Sadia Khurshid, Uzma Rasheed, Alam Zeb, Shazia Zammurrad

**Affiliations:** 1 Rheumatology, Pakistan Institute of Medical Sciences, Islamabad, PAK; 2 Radiology, Pakistan Atomic Energy Commission General Hospital, Islamabad, PAK

**Keywords:** juvenile idiopathic arthritis, depression, burden of disease

## Abstract

Background and objective

Juvenile idiopathic arthritis (JIA) is an idiopathic autoimmune rheumatic disorder in children. JIA has been associated with depression and has a negative psychological impact on patients' quality of life. The aim of the study is to determine the prevalence of depression in patients with JIA presenting at a tertiary care hospital in Islamabad, Pakistan.

Materials and methods

This cross-sectional study, conducted at the Department of Rheumatology, Pakistan Institute of Medical Sciences, Islamabad, Pakistan, included 100 children aged >6 years who had been diagnosed with JIA according to the 2004 revised International League of Associations for Rheumatology classification. Physical disability was measured using the Childhood Health Assessment Questionnaire disability index (CHAQ-DI), whereas depression was assessed by measuring their Center for Epidemiological Studies Depression Scale for Children (CES-DC) scores. Results were analyzed using IBM SPSS Statistics for Windows, Version 20.0. (Armonk, NY: IBM Corp.), with p-values ≤ 0.05 considered statistically significant.

Results

The 100 patients included 54 male patients (mean age, 16.3 ± 4.9 years) and 46 female patients (mean age, 18.6 ± 5.1 years). CES-DC scores showed that 72 patients with JIA had significant depression. Of these 72 patients, 50 (69.4%) had mild, 21 (29.2%) had moderate, and one (1.4%) had severe disability according to CHAQ-DI criteria. Age was the only effect modifier significantly associated with significant depression in patients with JIA (P < 0.05).

Conclusion

A cross sectional survey was carried out to find prevalence of depression in children with JIA. Physical disability and depression were measured using standardized tools. The percentage of significant depression among children with JIA is very high in our local population and was significantly associated with disease severity. Our findings emphasize the need to initiate early and prompt measures to prevent depression and reduce overall morbidity in patients with JIA.

## Introduction

Juvenile idiopathic arthritis (JIA) is one of the most frequent types of rheumatologic disorders in pediatric patients aged younger than 16 years [1]. JIA consists of a heterogeneous group of chronic disorders of unknown etiology encompassing all forms of arthritis. JIA is characterized by the non-infectious inflammation of synovial membranes of the joints and connective tissues that lasts for at least six weeks [2, 3]. Its global incidence has been reported to range from one to 23 per 100,000 persons per year, and its prevalence ranges from seven to 400 per 100,000 children [4-6]. Although the exact etiopathogenesis of JIA remains unclear, it may be due to immune dysregulation secondary to various environmental and genetic factors [7]. Some children experience the symptoms of JIA for a shorter time, whereas others experience its symptoms and their consequences for their entire lives [8].

During their course of the disease, children with JIA may also experience frequent episodes of depression and other psychosocial disorders [9, 10]. Depressive symptoms among these children may be conditioned by their restricted daily activities, altered body images, growth retardation, visual symptoms, limitations in leisure time, and frequent visits to the doctor [11]. Psychological problems, especially depression, are more prevalent in patients with JIA than in normal individuals, with the incidence of clinically significant depression ranging from 7% to 36% in children with JIA [12-14]. To further assess the incidence of this commonly occurring psychological disorder in patients with JIA, and to better understand the significant effect modifiers associated with the increased prevalence of depression in these patients, the present study assessed the burden of depression in JIA patients in the local population of Islamabad, Pakistan. The results of this study may guide rheumatologists to initiate early and prompt measures to prevent depression in this population, reducing overall morbidity in patients with JIA.

## Materials and methods

This cross-sectional study enrolled 100 children aged older than six years who fulfilled the 2004 revised International League of Associations for Rheumatology Classification for JIA and were evaluated at the Department of Rheumatology, Pakistan Institute of Medical Sciences (PIMS), Islamabad, Pakistan, from June 2018 to June 2019 [15]. Patients with comorbidities or other diseases causing arthritis and psychiatric problems, as well as those with Down’s syndrome or other cognitive problems, hearing and speech problems, trauma, sepsis, malignancy, bone diseases, dysplasia, or osteomyelitis were excluded. The design of this study was approved by the ethics committee of PIMS, and verbal and written consent was obtained from all JIA patients and their parents before study enrollment.

Physical disability was measured in these children using the Children Health Assessment Questionnaire with Disability Index (CHAQ-DI), with CHAQ-DI scores of 0 to 1, >1 to 2, and >2 indicating mild, moderate, and severe disability, respectively. Depression was evaluated using the Center for Epidemiological Studies Depression Scale for Children (CES-DC), a 20-item, self-reported inventory of depression with possible scores ranging from 0 to 60. Cumulative CES-DC scores <15 indicated no depression, whereas scores ≥15 indicated significant depression. Effect modifiers, including age, gender, eye involvement, disease duration, and presence of antibodies directed against the Fc portion of immunoglobulin G (rheumatoid arthritis factor - RAF) and antinuclear antibodies (ANA) detected via indirect immunofluorescence on Hep-2 cell lines controlled by stratification. We used post-stratification chi-square tests, with p-values <0.05 considered statistically significant.

## Results

The 100 patients included 54 male patients (mean age, 16.3 ± 4.9 years) and 46 female patients (mean age, 18.6 ± 5.1 years). Mean duration of disease in the total study population found to be 6.63± 4.67 years, while most of the patient (44%) was presented with less than five years of disease duration. Eye involvement was positive in 29% of the patients. Detailed demographic characteristics of the study population are summarized in Table 1 and Table 2.

**Table 1:** Demographic and clinical characteristics of the study population (n=100) JADAS - Juvenile Arthritis Disease Activity Score; CHAQ - Childhood Health Assessment Questionnaire; CES-DS - Center for Epidemiological Studies Depression Scale for Children

Variable	Mean	Standard Deviation (±)
Age (years)	17.39	5.09
Duration of disease (years)	6.63	4.67
Total no. of swollen joints	6.81	6.29
Total no. of tender joints	6.38	7.23
JADAS-27	19.92	13.81
CHAQ Score	0.60	0.51
CES-DS Score	23.64	12.70

**Table 2:** Frequency and percentages of demographic and clinical characteristics of the study population (n=100) RAF - rheumatoid arthritis factor; ANA - antinuclear antibodies

Variable		Frequency	Percentage
Gender	Male	54	54
	Female	46	46
Eye involvement	Positive	29	29
	Negative	71	71
RAF	Positive	19	19
	Negative	81	81
ANA	Positive	11	11
	Negative	89	89
Disease duration	<5 Years	44	44
	5–10 Years	37	37
	>10 Years	19	19
Age group	≤15 Years	35	35
	15–20 Years	34	34
	>20 Years	31	31

Study results illustrated that the mean CES-DC score in the total study sample was 23.64±12.70, while CHAQ-DI Score noted as 0.6031±.5150. As per recorded CES-DC score, it has been elaborated that 72 of the 100 patients with JIA had significant depression. Of these 72 patients, 50 (69.4%) had mild, 21 (29.2%) patients had moderate, and one (1.4%) had severe disability according to CHAQ-DI criteria, with analysis showing a statistically significant relationship between depression and severity of disease (p=0.004). Age was the only effect modifier that was significantly associated with significant depression in JIA patients (p < 0.05; Table 3).

**Table 3:** Stratification of depression in JIA patients on the basis of different sociodemographic factors RAF - rheumatoid arthritis factor; ANA - antinuclear antibodies; JIA - juvenile idiopathic arthritis

Socio-demographic factors		Significant depression		Total	p-value (Chi-square test)
		Absent	Present		
Gender	Male	19	35	54	0.083
		35.2%	64.8%	100.0%	
	Female	9	37	46	
		19.6%	80.4%	100.0%	
Eye involvement	Positive	7	22	29	0.583
		24.1%	75.9%	100.0%	
	Negative	21	50	71	
		29.6%	70.4%	100.0%	
RAF	Positive	2	17	19	0.059
		10.5%	89.5%	100.0%	
	Negative	26	55	81	
		32.1%	67.9%	100.0%	
ANA	Positive	4	7	11	0.513
		36.4%	63.6%	100.0%	
	Negative	24	65	89	
		27.0%	73.0%	100.0%	
Disease duration	<5 Years	14	30	44	0.674
		31.8%	68.2%	100.0%	
	5–10 Years	10	27	37	
		27.0%	73.0%	100.0%	
	>10 Years	4	15	19	
		21.1%	78.9%	100.0%	
Age group	≤15 Years	15	20	35	0.042
		42.9%	57.1%	100.0%	
	15–20 Years	8	26	34	
		23.5%	76.5%	100.0%	
	>20 Years	5	26	31	
		16.1%	83.9%	100.0%	

## Discussion

JIA is a not uncommon disorder in Pakistani children, with 60% to 80% of these children having polyarthritis and oligoarthritis, the most common subtypes of JIA [16, 17]. Social disturbances, pain, and deformities in children with JIA can cause psychological distress, with higher proportions of children and adolescents with JIA having significantly higher levels of depression than healthy individuals of the same age and gender [18-20].

The goal of this cross-sectional study was to assess the prevalence of depression in local children and adolescents with JIA and to evaluate the relationship of depression with disease severity and other effect modifiers. Studies worldwide have reported that children with JIA are prone to the development of psychosocial problems like depression. We found that the prevalence of significant depression in JIA patients was 72%, and that age group was the only effect modifier associated with significant depression. We also observed a statistically significant relationship between the degree of disease, as determined by CHAQ scores, and significant depression in this study population, a finding in agreement with a similar study in Egyptian children with JIA [21].

Complications faced by children with JIA in performing their daily school and recreational activities may cause them to feel different than their healthy peers, which may alter their mood. Although many studies have found that pain was significantly correlated with symptoms of depression in these patients, other studies have reported no significant relationship between depression and pain symptoms [21-23]. Moreover, significant depressive symptoms in children with JIA were found to be comparable to reference scores [24].

A cross-sectional study of the relationship between JIA and depression found that pain scores, disease duration, and CHAQ scores correlated significantly [10]. In contrast, our study did not show a statistically significant relationship between disease duration and depression. A study analyzing the long-term impact of JIA on the psychosocial life of a Greek population showed that disease activity correlated significantly with a higher degree of depression (p = 0.032) [25].

The level of depression in the present study population was higher than that in previous studies. For example, a long-term follow-up study of patients with JIA found that the incidence of depression was only 5.2% [26], with another study finding that depression was present in 31% of JIA patients [27]. In contrast, this study reported significant depression in 72% of children with JIA.

The main strength of this study was its evaluation of the association between depressive symptoms and JIA in a pediatric population in a developing country. However, this study had several limitations, including the relatively small number of patients, with most of the population belonging to the same geographic and ethnic group. Large-scale studies that include patients at multiple centers throughout the country are required.

## Conclusions

We carried out a cross-sectional survey to determine the prevalence of depression in children with JIA, at a tertiary care hospital. Physical disability and depression were measured using standardized tools. A high percentage of children with JIA were found to have depressive symptoms, which were significantly associated with the severity of JIA. Depression was significantly associated with age group but not with gender, eye involvement, RAF, ANA, or disease duration. These results emphasize the need to initiate early and prompt measures to prevent depression in this population, and in reducing overall morbidity in patients with JIA.
